# Avoiding fears and promoting shared decision-making: How should physicians inform patients about radiation exposure from imaging tests?

**DOI:** 10.1371/journal.pone.0180592

**Published:** 2017-07-07

**Authors:** Blanca Lumbreras, José Vilar, Isabel González-Álvarez, Mercedes Guilabert, María Pastor-Valero, Lucy Anne Parker, Jorge Vilar-Palop, Ildefonso Hernández-Aguado

**Affiliations:** 1 Department of Public Health, History of Science and Gynecology, Miguel Hernández University, Alicante, Spain; 2 CIBER en Epidemiología y Salud Pública, Madrid, Spain; 3 Radiodiagnostic Department, Peset Hospital, Valencia, Spain; 4 Radiodiagnostic Department, San Juan Hospital, Alicante, Spain; 5 Psychology department, Miguel Hernández University, Alicante, Spain; 6 Centro Nacional de Dosimetría - Radonic y CRC, Valencia, Spain; Centro per lo Studio e la Prevenzione Oncologica, ITALY

## Abstract

**Objective:**

We aimed to evaluate the population’s awareness about the radiation exposure associated with five specific imaging tests, and their preference regarding three different formats for receiving the information before undergoing an imaging test.

**Methods:**

A quantitative and qualitative evaluation through a survey and focal groups including general population from two health departments in Spain. The sampling was carried out in stages (according to health department size) and stratified by age and sex, to get a representative sample. We randomly selected the participants from these stages to be contacted by telephone by a trained nurse. Oral informed consent was obtained.

**Results:**

Of 602 participants in the quantitative survey, 418 (70.3%) stated that they were aware of the risk associated with radiation. While the majority of these 418 participants knew that x-rays involve radiation (85.4%), fewer were aware that CT (42%) and mammography (38%) also involve radiation, and a substantial proportion believed, incorrectly, that MRI (38%) and ultrasound (18.4%) expose patients to radiation. The population preference was to receive the information using both oral and written formats, accompanied by a table showing the equivalence of the radiation associated with the imaging test to either a number of chest X-rays and exposure number of days of background radiation.

**Discussion:**

The general population does not receive enough information regarding radiation exposure and the associated risks related to imaging tests. Initiatives should be designed to reinforce the patient’s awareness when ordering a diagnostic imaging test.

## Introduction

Healthcare overuse is an increasing problem with adverse consequences for patients and physicians, and serious economic implications [1]. Several attempts to deal with this problem have been made, most of which primarily involve healthcare providers. However, there is a general consensus that any strategy should also include the individual patient’s opinion and preferences [2].

In the last decades, a significant part of healthcare overuse is attributable to a massive increase in medical imaging [3]. This increase, mainly observed in Computerized Tomography (CT), has led some institutions such as the Food and Drug Administration [4] and the European Commission [5] to introduce legislation to prevent overuse of medical imaging and avoid unnecessary radiation exposure to the population. One of their main initiatives is to enforce the recording of the radiation dose received by each patient undergoing a medical imaging test [5]. This will increase physicians’ awareness of the risks associated with the imaging tests. It is also necessary that patients understand the risks of radiation associated with imaging tests, given that previous studies have shown that well informed patients are less likely to request unnecessary diagnostic tests [6]. At the same time, patients need to be informed about the risks associated with not undergoing a specific imaging test as well.

To accomplish this, physicians need to be aware of how much patients know and how they feel about radiation exposure. Nevertheless, few studies have been conducted to assess patient knowledge of and attitudes towards radiation [7–10] and only some have focused on the general population [11]. Additionally, these studies showed that although CT is estimated to be responsible for more than 70% of the collective radiation dose received by patients, most of them lack information about the reasons behind the imaging test prescribed (the benefits) and the associated radiation dose (the risks).

Successfully communicating the benefits and risks of medical imaging tests to patients is related to which information is provided and the way it is presented, both of which should be adapted to the patients’ capacity [12]. Several approaches aiming to reduce unnecessary imaging tests in clinical practice have been discussed [13]. Given the population’s lack of familiarity with radiation terminology, such as effective dose or milliSievert (mSv), several authors compared these concepts to other, more familiar ones [14]. For instance, the most common comparisons for communicating radiation exposure are the equivalent number of chest X-rays, the equivalent number of days of background radiation or the equivalent number of transoceanic flights [12]. However, to our knowledge, no studies have analyzed patients’ preferences for different presentation formats comparing these approaches when communicating risks associated to radiation exposure.

The aim of this study was to evaluate the general populations’ awareness about the radiation exposure associated with five specific diagnostic imaging tests, and their preference regarding three different formats for receiving the information before undergoing a medical imaging test.

## 2. Materials and methods

### 2.1 Design

We carried out a quantitative and qualitative evaluation through a survey and focal groups to achieve a comprehensive picture of patients’ understanding of the benefits and risks associated with medical imaging and their opinions about how this type of information should be delivered.

### 2.2 Quantitative study

#### Participants

The target population consisted of the residents in the catchment area of two public hospitals in the Autonomous Community of Valencia, Spain: Dr Peset Hospital (Health Department number 10) and San Juan Hospital (Health Department number 17). The catchment populations were 377,780 and 234,424 people, respectively. The two hospitals belong to the Spanish National Health Care System and are referral hospitals for all individuals living in the geographic area. The majority of the population in these areas uses the National Health Care System as the main medical service.

#### Sample size estimation and selection of participants (Fig 1)

**Fig 1:**
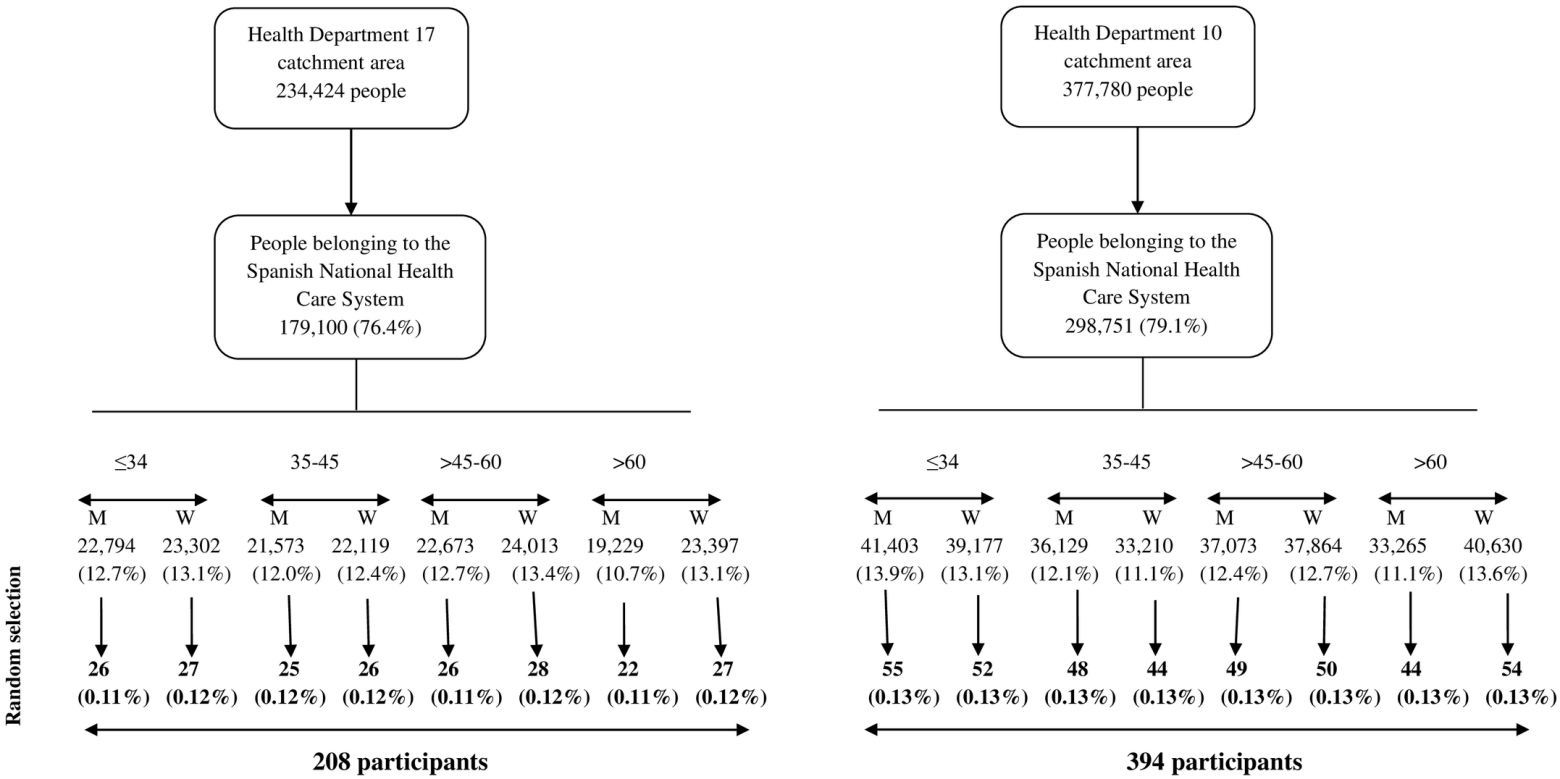
Flow diagram of selection procedure.

We estimated that, for a precision of 5% with 95% confidence intervals, at least 384 people would be required to estimate the proportion of individuals that know that imaging tests incur a health risk based on a conservative estimate that 50% of the population could be unaware of the radiation exposure associated with imaging tests. To allow for analysis by subgroups we increased this number to 602.

A complete list of the catchment populations from both Health Departments was obtained with names, date of birth and phone numbers (this was done in strict adherence to current regional (Autonomous Community of Valencia) regulations). The sampling was carried out in stages (according to health department size) and stratified by age (≤34; 35–45; 45–60; >60 years old) and sex. We randomly selected in each category the participants to be contacted until we reached the target number for enrolment. The participants were contacted by telephone and invited to participate, and oral informed consent obtained by a trained nurse from the radiological departments.

#### Survey design

We developed a questionnaire *ad hoc* that included the following items: 1) socio-demographic characteristics (age and sex) of participants; 2) if they had had an imaging test in the previous 12 months and the type of imaging test; 3) their knowledge regarding the risks associated with radiation exposure from imaging tests; 4) their feelings about the information regarding the risks associated with medical imaging, and 5) if providers discussed the imaging test procedure, including risks, benefits and indications for performing an imaging test (S1 Text). The questionnaire was piloted by a number of nonmedical staff prior to use. The pre-piloted survey was answered by 4 people. After the pilot, the question ‘What do you think about the information you receive from the physician?’ was transformed into multiple choice to facilitate answering and analysis of the questionnaire (at the beginning this question only had two possible answers, a) easy to understand or b) difficult to understand). This modified questionnaire was piloted in a different sample of 4 people.

#### Statistical analysis

Basic descriptive statistics were obtained for each question using SPSS 22.0 (IBM). Cumulative frequency and percentage values for all responses were estimated.

The outcome measures were: referred awareness of radiation exposure associated with imaging tests; awareness of the radiation exposure associated with particular imaging tests; ability to share with the physician the decision to prescribe the imaging test; and preferred format to receive information about risks of radiation exposure associated with imaging tests. These questions were quantified according to the answers shown in the survey.

Associations between groups were analysed using the Pearson Chi^2^ test, with p < 0.05 considered statistically significant. The effect of diverse explicative variables was considered by means of a stratified analysis and unconditional logistic regression was used (95% confidence intervals). A multivariate logistic regression model was built applying a stepwise procedure to enter variables in the model.

### 2.3 Qualitative study

#### Participants

Two focus groups were conducted separately in the two participating hospitals; one in San Juan de Alicante Hospital and one in Doctor Peset Hospital in Valencia during October and November 2015.

Participants were selected from those included in the survey who expressed their willingness to participate in a focus group. From those, we randomly selected people between 18 and 90 years old and representative of three age groups: <30 years old, 30–50 years old and more than 50. Selected participants were contacted by the researchers, informed about the aim of the study and invited to join the focus groups.

#### Procedure

The two groups used an identical protocol and procedure, which began with a short presentation by the head of the radiology department in each hospital and all participants being informed and consenting to the study. The focus group discussions lasted between 60–90 minutes and were audio recorded.

The research team developed a semi-structured focus group protocol to guide the discussion based on a literature review of radiation exposure topics. The protocol was divided into three main themes: a) Assessment of the information they receive before having an imaging test; b) what information they would like to receive before undergoing an imaging test, and c) the participants assessed three potential information sheets detailing the radiation exposure risk associated with specific imaging tests to determine which they felt would be easiest to understand. These information sheets (S2 Text and S1 Fig) were: 1) the official information given in current clinical practice in these hospitals; 2) an adapted radiation equivalence table [12], showing the effective radiation dose received by the different imaging tests under study expressed as radiation exposure units (u) equivalent to one chest X-ray. The table also showed the radiation equivalence of each test corresponding with one year’s natural background radiation exposure in different geographical locations, and 3) a figure showing a visual representation of medical radiation exposure of each imaging test (compared to background radiation exposure), this last one designed by the authors.

#### Data analysis

Participants’ identification code numbers were de-identified by replacing the original code number with a new random code number.

Demographic data were summarized for all study participants using descriptive statistics. Audio-recordings were transcribed literally and notes from the interviewers were used for later analysis. All personal identifiers were removed.

First, a careful transcription reading was made and the text then split up into meaningful information units. These units were coded following a mixed strategy (emerging and predefined codes according to the study objectives), and categories were developed on the basis of grouping codes with the same topic.

Finally, the points of agreement and disagreement were analysed and triangulation of the results were performed to qualitatively analyse the degree of agreement.

### Ethics statement

Oral informed consent was obtained from participants in the quantitative survey (because they were contacted by phone) and written informed consent from those participants in the focus group. Institutional Review Board approval was obtained from San Juan Hospital Committee (Ref 14/301) and Dr Peset Hospital Committee (Ref 2/14).

## 3. Results

### 3.1 Quantitative study

Out of 662 people who were initially selected to be included in the study, we were unable to contact 50 (7.5%) of them on the phone and 10 (1.5%) declined to answer the survey.

A total of 602 people were included in the quantitative study (Fig 1): 208 (34.6%) from Health Department 17 and 394 (65.4%) from Health Department 10. The mean age was 53 (IQR 40–66) years old and 334 (55.5%) were women. There were not demographic differences between the two health departments.

#### Awareness of radiation exposure associated with imaging tests

418 (70.3%) participants stated that they were aware of the risk associated with radiation in imaging tests. Awareness was higher among women (249/334, 74.6% for women vs. 169/268, 63.1% for men, p = 0.008) and in Health Department 17 (165/208, 79.3% for HD 17 vs. 253/394, 64.2% for Health Department 10, p<0.001). People who reported that they had been informed about the risks of the imaging test they had been prescribed were more likely to report that they knew about radiation exposure (79/102, 77.4%, vs 323/475, 68.0% p = 0.048) (Table 1).

**Table 1:** Population awareness about the risk associated with imaging test and related variables.

Variable (N;%)		Total	Are you aware of the risks associated with radiation exposure in imaging tests?		*p value*
			No (177; 29.7%)	Yes (418; 70.3%)	
**Sex**					*0*.*008*
	Men	268	96 (35.8)	169 (63.1)	
	Women	334	81 (24.3)	249 (74.6)	
**Age (years)**					*0*.*291*
	≤35	92	30 (32.6)	60 (65.2)	
	35–45	114	29 (25.4)	84 (73.7)	
	>45–60	175	43 (24.6)	131 (74.9)	
	>60	221	75 (33.9)	143 (64.7)	
**Health Department**					*<0*.*001*
	10	394	141 (35.8)	253 (64.2)	
	17	208	36 (17.3)	165 (79.3)	
**Have you had an imaging test in the last 12 months?**					*0*.*232*
	No	276	92 (33.3)	180 (65.2)	
	Yes	278	75 (27.0)	200 (71.9)	
**Did the physician inform you about the benefits of the imaging test and why you are having it?**					*0*.*004*
	No	273	93 (34.1)	173 (63.4)	
	Yes	297	78 (26.3)	219 (73.7)	
**Did the physician inform you about the risks associated with imaging tests involving radiation?**					*0*.*048*
	No	475	145 (30.5)	323 (68.0)	
	Yes	102	23 (22.5)	79 (77.5)	

#### Assessment of the information received when undergoing an imaging test

Of the 102 people who were informed about risks of the imaging test by the physician, 56 (54.9%) shared the decision to order these tests with their physician (Table 2).

**Table 2:** Assessment of the information received when undergoing an imaging test and the ability to share the decision with the physician to ask for a specific imaging test.

Variable (N; %)		Total (102; 100.0)	Does the information you receive enable you to share the decision with the physician regarding whether to order an imaging test?			*p valor*
			No (34; 33.3)	Yes (56; 54.9)	Unknown (12; 11.7)	
**What type of information do you receive from the physician?**						*<0*.*001*
	Oral	29	17 (58.6)	11 (37.9)	1 (3.4)	
	Written	46	13 (28.3)	31 (67.4)	2 (4.3)	
	Both	21	4 (19.6)	14 (66.7)	3 (14.3)	
	Unknown	6			6 (100.0)	
**How much information do you receive from the physician?**						*<0*.*001*
	Very little	78	30 (38.5)	44 (56.4)	4 (5.1)	
	Sufficient	11	4 (36.4)	7 (63.6)		
	A lot	4		4 (100.0)		
	Unknown	9		1 (11.1)	8 (88.8)	
**What do you think about the information you receive from the physician?**						*<0*.*001*
	Difficult to understand	3	2 (66.7)	1 (33.3)		
	Can be understood with some difficulty	15	10 (66.7)	5 (33.3)		
	Easy to understand	67	19 (28.4)	45 (67.2)	3 (4.5)	
	Very easy to understand	7	2 (28.6)	5 (71.4)		
	Unknown	10	1 (10.0)		9 (90.0)	
**The effect of the information you receive is:**						*<0*.*001*
	I do not trust it	1	1 (100.0)			
	It has no special effect on me	41	25 (61.0)	15 (36.6)	1 (2.4)	
	It reassures me	51	8 (15.7)	40 (78.4)	3 (5.9)	
	Unknown	9		1 (11.1)	8 (88.8)	

The proportion of participants who reported that they were able to share the decision to order the imaging test was higher among those who reported that they had received either written (31/46, 67.4%) or oral and written information (14/21, 66.7%) compared to those who only received oral information (11/29, 37.9%) (p<0.001). Similarly, people who reported that they had very little information were less likely to report shared decision making (44/78, 56.4%) compared to those that reported they had received a lot (4/4, 100.0%) or sufficient information (7/11, 63.6%, p<0.001).

Furthermore, participants who thought that the information they received was easy to understand (45/67, 67.2%) or very easy to understand (5/7, 71.4%) were more likely to report that they shared the decision with the physician compared to those that thought that the information was difficult to understand (1/3, 33.3%) or could be understood with some difficulty (5/15, 33.3%) (p<0.001). Finally, shared decision making was more common among participants who reported that information regarding the radiation exposure associated with medical imaging reassured them (40/51, 78.4%) compared to those who felt that it had no special effect on them (15/40, 37,5%) (p<0.001).

#### Knowledge regarding doses and risks related to medical imaging

**Fig 2:**
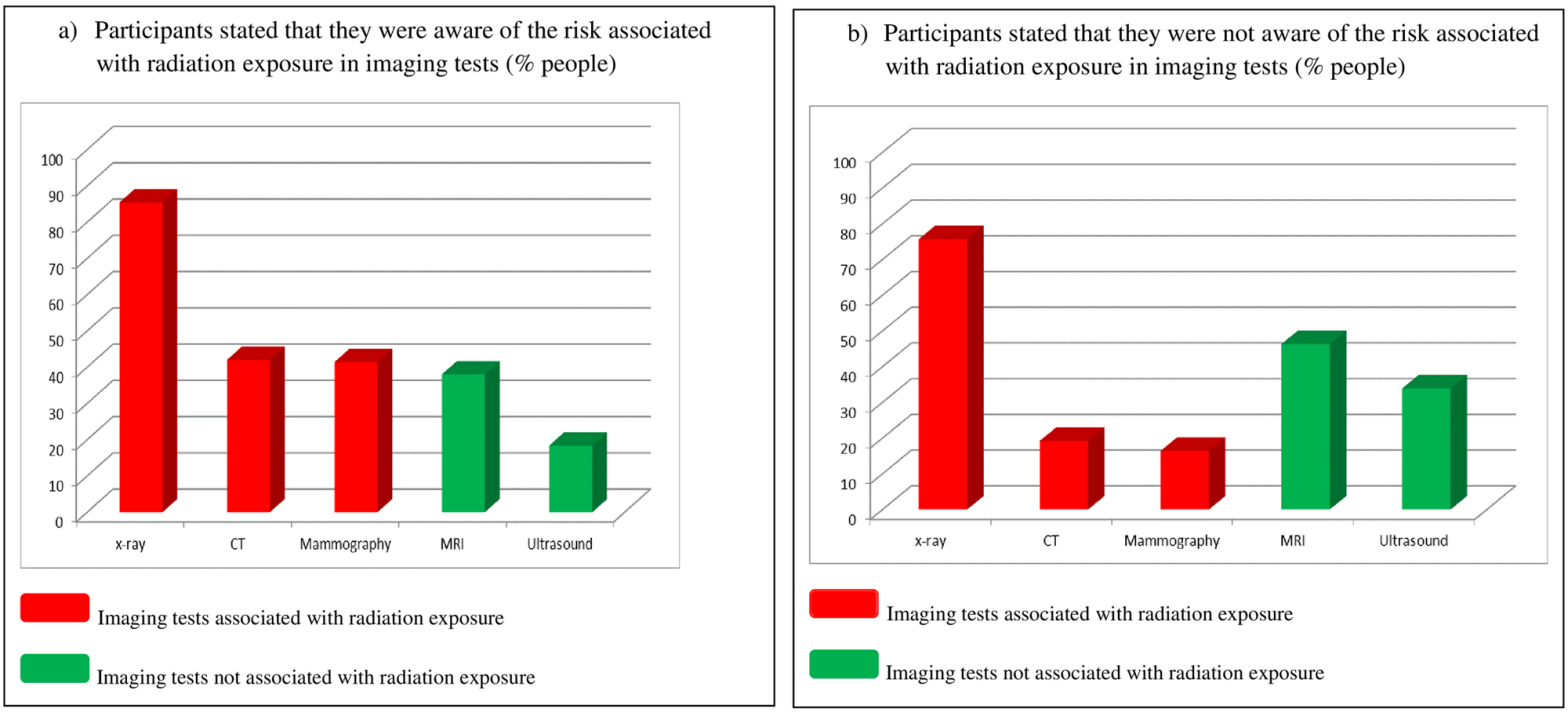
Percentage of people that associated each particular imaging test with radiation exposure according to that they were awareness of the risk associated with radiation exposure in imaging tests. a. Participants stated that they were aware of the risk associated with radiation exposure in imaging tests. b. Participants stated that they were not aware of the risk associated with radiation exposure in imaging tests.

While the majority of the 418 participants who stated that they were aware of the risk associated with radiation in imaging tests knew that x-rays emit radiation (85.4%), fewer were aware of the radiation emitted by CT (42%) and mammography (38%) and a substantial proportion believed that MRI (38%) and ultrasound (18.4%) had radiation. Of the 177 participants who stated that they were not aware of the risks associated with radiation exposure in imaging tests, 75.7% knew that x-rays involve radiation, 19.2% and 16.4% knew that CT and mammography respectively, had radiation, 46.3% and 33.9% believed that MRI and ultrasound respectively, had radiation (Fig 2). Thus, patients who reported that they were aware of the risks of radiation associated with medical imaging were barely able to identify which specific tests emit radiation any better than those who reported that they were unaware of the risks (p = 0.342).

### 3.2 Qualitative study

Out of 72 people invited to participate in the focal groups, 20 people participated in the two focal groups (27.7%), 12 women (60%) and 8 men. The results are grouped according to the three main proposed topics.

#### a. Assessment of the information received before undergoing an imaging test

Overall, the interviewed population stated that they received little information regarding the radiological test they were going to have:

‘*They hardly ever explain anything; only the place and time*’.

‘*I have a mammography every two years, but they do not give me any information at all; they only tell me where to go and that is it*’.

‘*Sometimes, I wonder why they have asked for that particular test*’.

They also stated that several times the professional only informs them about pre-test procedures:

‘*I was given an information sheet telling me how to prepare myself and not to eat before the test, and I have never received any more information*’.

‘*The same happened with the CT that I had: they explained that they were going to inject me with a liquid to get a contrast, so that they could see the image they needed to make a diagnosis*’.

However, few of the participants felt the need to ask the physicians for more information.

‘*I think that if they have asked for the test it is because it will help them to make a better diagnosis; since it does not hurt, I do not think about it much*’.

‘*I think we often just trust the doctor. When my son was operated you put yourself in the doctors’ hands and if he asks for an X-ray he must have a reason*’.

Most of them stated that they did not know enough about radiation exposure terms to understand the physicians’ explanation:

‘*Sometimes, they use a lot of technical terms and when you try to read them you can’t understand a thing*’.

Some individuals commented that on a few occasions they were informed about the associated risk for specific imaging tests, especially those not involving radiation.

‘*I always wonder how much radiation I am going to receive because I have never studied it and nobody has ever told me. However, I do know that MRI does not have*’.

Despite this lack of specific information regarding the risks, most of them agreed to sign the written informed consent especially as it was delivered to them just before the test was performed, which could be perceived as being too late to say no.

‘*We were asked to sign the informed consent just before the test, when the child was already sedated*’.

#### b. What information would participants like to receive before undergoing an imaging test?

Participants stated that communication between health professionals regarding tests was limited. They also pointed out the importance of keeping count of the number of tests carried out on each patient.

‘*Yes, it sometimes happens. A doctor can ask for a new test without realizing that you already had one. Physicians should have a record of the imaging tests received by each patient*’.

‘*It would be very interesting if physicians provide us an annual or monthly register including the number of imaging tests we have had*’.

Interviewed participants preferred both verbal and written information, describing the benefits and risks of diagnostic imaging tests. Furthermore, they underlined the importance of receiving information in a summarized form:

‘*If you only receive written information, when you are in your house and try to understand it, you cannot clarify any questions with the physician*.

The majority of the participants preferred to receive limited information:

‘*I do not usually read long informative texts… I think the patient should receive a minimum amount of information with the possibility of extending it if you want*’

Participants wanted to know the purpose and a brief description of the test.

‘*I would like to have a brief description of the test I am going to have*’.

‘*I think that to know what they are going to discover with the test is a priority*’.

The participants´ pathologies also influence their opinions. In general, the older participants who had had cancer or were currently receiving treatment for cancer said that they did not need this information because they trusted the physician’s decision.

‘*If the physician thinks that I need the test to improve the management of my disease, I consider that the benefit/risk balance is in my favour*’.

Nevertheless, younger participants were more concerned about the long term-risk associated with imaging tests and asked physicians for a detailed description about the associated risks. However, some of them had misconceptions about radiation exposure:

‘*I would like to know the amount of radiation I receive when I have a particular imaging test and what are the long-term effects of this radiation in my body*’.

‘*I need to know the benefit/risk of having a CT and the amount of time I have to wait until I receive a new CT to avoid the accumulation of the radiation exposure*’.

Participants requested that physicians take into account the patients’ emotional state when explaining the diagnostic test as it increased their anxiety.

‘*Health professionals need to be aware that when they inform patients about a medical procedure, the patient could be in shock and he/she only receives a part of the information*’.

#### c. Evaluation of the three potential formats to explain risks associated with imaging tests

All of the participants agreed that the most appropriate way to present information was a table showing a number of imaging tests and their corresponding radiation equivalence in terms of chest X-rays and background radiation exposure.

## 4. Discussion

This study highlights the lack of knowledge in the general population and the limited information received from the health professionals regarding the radiation exposure associated with five different diagnostic imaging tests.

Although more than 70% of the participants affirmed that they were aware of the risks associated with radiation exposure in imaging tests, only 30% of them knew that CT or mammography involve radiation. Moreover, 38% of the participants thought that MRI involves radiation. In the qualitative study, most of the participants stated that they did not know enough radiation exposure terms to understand the physicians’ explanation and some of them had misconceptions about radiation exposure that could alter their expectations of benefits versus risks.

Previous studies have also shown that patients frequently do not know about the risks involved in imaging tests [8–10]. However, a recent survey in USA^14^ showed an increase in patients’ knowledge, with 70% being aware that CT scans involve radiation exposure and 25% knowing that radiation can increase overall lifetime risk of cancer. In contrast, another study, which was also carried out in a public environment showed a low level of awareness of the implications of radiation exposure [11]. The present study represents the first survey in Spain and therefore we cannot compare it with previous national data. The previous studies were carried out in clinical settings where the fact that patients were waiting to undergo a medical procedure may have introduced unintended bias. In contrast, our study includes the general population of whom less than half had had an imaging test in the previous 12 months. Thus, some factors such as the priority of the immediate health problem over the future development of cancer associated with imaging tests could have had less influence on their answers.

In this study some factors were unexpectedly associated with a greater awareness of the risks associated with imaging tests: gender and the health department. However, in previous studies where awareness and demographics were evaluated [7], no gender differences were shown. One potential explanation for higher awareness among female participants could be that in Spain there is a high adherence to the breast cancer screening program, and this, might have contributed to a better awareness about radiation associated with imaging test. We also found differences in awareness of risks and benefits associated with the health department from which the participant was from, although both centres used the same informed consent. These findings were unexpected but are in line with previous studies, where there was also substantial variation in patients’ perceptions across sites [15].

Less than 20% of the interviewed population indicated that the physician informed them about the risks associated with imaging tests involving radiation. Similarly, in the qualitative evaluation, the participants stated that they received little information regarding the radiological risks and they pointed out that they only received information about how they should prepare themselves for the test.

Conversely, in the quantitative survey, those who were informed by the physicians stated that they had a greater awareness of the risks associated with radiation exposure in imaging tests. Previous studies also showed that physicians do not explain radiation exposure involved in diagnostic imaging studies to patients as a result of their own low awareness level regarding radiation risks [16]. One of the key innovations in the revised ‘Basic Safety Standards Directive’ adopted in 2013 by all European member states [5] is the need to inform about and, if appropriate, record the radiation dose received by each patient undergoing a medical imaging test. In line with this, the participants of the qualitative study emphasized the importance of keeping count of the number of tests carried out on each patient to allow the physicians to control the cumulative dose received.

Although there were no differences in awareness of the risks associated with radiation exposure according to participants’ age in the quantitative survey, there were differences in participants’ attitudes according to their age in the qualitative evaluation. Younger patients were more concerned about the long term-risk associated with imaging tests compared with older patients with a chronic disease who did not feel the same need to ask the physicians for more information. Therefore, not all patients wanted to have more information about radiation, and some of them were happy to adopt a more passive role. Other authors have previously reported that patients with a low risk illness and younger patients are generally more concerned about the use of imaging tests [1].

Communication regarding associated risk is essential in order to achieve a rational use of diagnostic imaging tests. However, the manner in which communication between physicians and patients is performed is also important [12]. Previous studies have shown that a single written decision support sheet is not enough to allow patients to share the decision of having an imaging test with their physicians [17]. In this study, patients who received only written or both oral and written information acknowledged that they were more able to share the decision regarding whether to order an imaging test compared to those who only received information orally. In line with this, participants of the focus groups preferred both verbal and written information describing the benefits and risks of diagnostic imaging tests.

On the other hand, the survey revealed that most of the respondents felt that they received very little information although this information was easy to understand. However, the participants of the qualitative study highlighted the use of technical terms by physicians and the importance of receiving comprehensive information in a summarized format. Thus, when participants received comprehensive information about radiation exposure from diagnostic imaging tests they appeared to be more likely to share the decision with the physician regarding whether to order the imaging test or not.

This study also evaluated patients’ preferences for different formats of presenting the risks involved with radiation exposure in diagnostic imaging tests. When participants were asked about the best written format to explain the risks related to radiation, the equivalent number of chest X-ray procedures and the equivalent time exposed to background radiation were the preferred formats.

Physicians are encouraged to reinforce the role of the patient when deciding whether to order a new imaging test. For this to happen, it is essential to increase patient’s knowledge on benefit/risk associated with imaging tests, and to allow an informed discussion with patients, taking into account the patient’s emotional state. Some tools could help to improve this communication, such as the format chosen in this study by the participants, or the incorporation of a patient’s radiation dose history that physicians could check before ordering a new diagnostic imaging test [18].

Although the risk associated to ionizing radiation from medical imaging tests has focused the attention of clinicians and stakeholders, a debate exists on this point. The level of exposure associated to these tests is very low and the risks of low-level radiation exposure remain controversial. There is a risk which increases with greater exposure, repeated exposures and with certain patient groups such as young patients, which is called ‘the deterministic effect’. High levels of radiation exposure can affect tissue (such as skin burns and depilation). In contrast, ‘the stochastic effect’ is random; the occurrence of individual events cannot be predicted. Stochastic effects can be divided into two groups, genetic and carcinogenic, where there is no threshold dose above which these effects will definitely occur or below which the likelihood of these effects could be zero.

The exposure to imaging tests involves a finite stochastic risk with a very long latency period, but there are clearly risks associated with not performing a test that should be carried out. Therefore, this is the main challenge in communicating risk associated with medical exposures. However, given that associated risks are cumulative, there are special situations to be taken into account. For instance, there is great debate about this topic in imaging test-based screening such as breast cancer screening, where cumulative radiation-induced cancer risks is weighed against the program's lifetime benefits, as opposed to an isolated screening test [19]. The generalizability of the results must be considered with due caution given that we only had two recruitment areas and that the Spanish population can be different from others. However, we selected a representative sample from the general population stratified by age and sex. Moreover, the survey targeted as many as 90% of subjects in the population sample, minimizing the risk of selection bias. This high acceptance rate could be due to the characteristics of the survey (obtained by phone by a trained nurse from the radiological departments). In contrast, although the participants had agreed to participate in the focal groups, in the end only 27.7% of them attended in person.

There were limitations in the study. We used a previously published chart comparing radiation doses and calculated risks of several imaging tests to other tests and other sources of radiation [12]. However, while this approach could help patients to understand the associated radiation dose, it is much more difficult to comprehend the associated risk. We created another chart comparing radiation exposure to risks related to other common activities (such as background exposure or the flight Madrid-New York) to try to solve this problem.

The survey was not previously validated. However, we piloted it in a different sample to check its feasibility to be used.

## 5. Conclusions

In conclusion, this study highlights the lack of knowledge in the general population and the limited information delivered by the health professionals regarding the risks associated with radiation exposure from imaging tests. Initiatives should be designed to reinforce patients’ awareness of radiation exposure and their role when ordering a diagnostic imaging test. Some tools could help, such as a table detailing the radiation equivalence in terms of x-rays, of background radiation, or of associated cancer risk, or the availability of the patient’s radiation dose history.

## Supporting information

S1 Text Survey. (DOC)

S2 Text Information sheets to be given to patients detailing the radiation exposure associated with imaging, which were evaluated by the clinician participants. (DOCX)

S1 Fig A figure showing a visual representation of the medical radiation exposure (compared to background radiation exposure). (JPG)
