# Supplementary material for: Avoiding fears and promoting shared decision-making: How should physicians inform patients about radiation exposure from imaging tests?
Source: PLoS One. 2017 Jul 7;12(7):e0180592. doi: 10.1371/journal.pone.0180592 (PMC5501589; doi:10.1371/journal.pone.0180592)
Supplement: S1 Text — (DOC) [file pone.0180592.s001.doc]

**Supplementary text 1: Survey:**

**The following survey aims to assess the health professionals’ knowledge about the radiation risk associated with imaging tests, as well as knowledge of the available recommendations. Please complete the sections of the entire survey and if you have any comment, you can fill in the comments section at the end of it.**

**Identification data:**

- Sex:

- Age:

**- Health Department:** 10 () 17 ()

**1- Are you aware of the risks associated with radiation exposure in imaging tests?**

Yes ( ) No ( )

**If yes, do you know the type of risks associated?**

**______________________________________________________________________**

**2- Check which one of the following tests is associated with radiation exposure:**

X-ray ( ) Ultrasound ( ) Magnetic resonance imaging ( ) Mammography ( ) CT ( )

**3- Have you had an imaging test in the last 12 months? Which one? ______________________________________________________________________**

**4- Did the physician inform you about the risks associated with imaging tests involving radiation?**

Yes ( ) No ( ) Who? **_______________________**

**If yes:**

**4.1 Type of information given:**

Oral () Written (informed consent () Both ()

**4.2 Amount of information given:**

Very little ( ) Sufficient ( ) A lot ( ) )

**4.3** **What do you think about the information you receive from the physician?**

Difficult to understand ( ) Can be understood with some difficulty ( ) Easy to understand ( ) Very easy to understand ( )

**4.4** **The effect of the information you receive is:**

I do not trust it () It has no special effect on me () It reassures me ()

**4.5.** **Does the information you receive enable you to share the decision with the physician regarding whether to order an imaging test?**

Yes ( ) No ( )

**5- Did the physician inform you about the benefits of the imaging test and why you are having it?**

Yes ( ) No ( ) Who? **_______________________**

**Observations:**
