# Supplementary material for: Avoiding fears and promoting shared decision-making: How should physicians inform patients about radiation exposure from imaging tests?
Source: PLoS One. 2017 Jul 7;12(7):e0180592. doi: 10.1371/journal.pone.0180592 (PMC5501589; doi:10.1371/journal.pone.0180592)
Supplement: S2 Text — (DOCX) [file pone.0180592.s002.docx]

**Supplementary text 2: Information sheets to be given to patients detailing the radiation exposure associated with imaging, which were evaluated by the clinician participants.**

**a) The official information given in current clinical practice in these hospitals.**

*Most frequently associated risks*

*Irradiation:*

*A CT is associated with ionizing radiation (x-rays) so it should be avoided in the case of pregnant women. In the rest of the population, the CT is only carry out when there is a precise indication to do it, because it has associated a high amount of radiation exposure.*

*As a guideline it should be noted that the dose received by the patient with the practice of a Skull CT scan radiation (2.3 mSv) is equivalent to 115 chest X-rays and is similar to 1 year of background radiation. Spiral CT (8mSv) radiation is equivalent to 400 chest X-rays and 3.5 years of background radiation. Abdominal CT scan is equivalent to 500 chest X-rays and 4.5 years of background radiation.*

*The potential risk of radiation includes a slightly elevated risk of cancer within a few years. This risk is less than 0.5%, so it can be considered very low compared to the normal incidence of cancer in the population, which is 33% for women and 50% for men, according to the American Society of Cancer.*

**b) An adapted radiation equivalence table^7^, showing the effective radiation dose received by the different imaging tests under study expressed as radiation exposure units (u) equivalent to one chest X-ray.**

*Most frequently associated risks*

*Irradiation:*

*A CT is associated with ionizing radiation (x-rays) so it should be avoided in the case of pregnant women. In the rest of the population, the CT is only carry out when there is a precise indication to do it, because it has associated a high amount of radiation exposure.*

*As a guideline, the following table shows the equivalence between different imaging tests. For instance, the skull CT, with a radiation dose associated of 2.3 mSv, is equivalent to 115 chest x-rays and 1 year of background radiation (a person is exposed to 2.4 mSv of background radiation by year). The risk of cancer associated is from 1/ 100.000 to 1/10.000 (which is 33% for women and 50% for men, according to the American Society of Cancer)*

| **Imaging test** | **Effective dose (mSv)** | **Chest x-rays equivalent** | **Background Equivalent Radiation Time** |
| --- | --- | --- | --- |
| Chest x-rays | 0.02 |  | 3 days |
| Skull CT | 2.3 | 115 | 1 year |
| Chest CT | 8 | 400 | 3.6 years |
| Abdomen CT | 10 | 500 | 4.5 years |

**c) A figure showing a visual representation of the medical radiation exposure (compared to background radiation exposure) (S2 figure).**

*Most frequently associated risks*

*Irradiation:*

*A CT is associated with ionizing radiation (x-rays) so it should be avoided in the case of pregnant women. In the rest of the population, the CT is only carry out when there is a precise indication to do it, because it has associated a high amount of radiation exposure.*

*As a guidelines, the following graphs shows the equivalences between the radiation absorbed by each imaging test and other radiation sources, according to the long-term potential risk: low (green), medium (yellow) and high (red):*
